# Damage to posterior parietal cortex impairs two forms of relational learning

**DOI:** 10.3389/fnint.2012.00045

**Published:** 2012-07-12

**Authors:** Siobhan Robinson, David J. Bucci

**Affiliations:** Department of Psychological and Brain Sciences, Dartmouth College, HanoverNH, USA

**Keywords:** medial temporal lobe, attention, associative learning, conditioned inhibition, parietal cortex

## Abstract

The posterior parietal cortex (PPC) is a component of a major cortico-hippocampal circuit that is involved in relational learning, yet the specific contribution of PPC to hippocampal-dependent learning is unresolved. To address this, two experiments were carried out to test the effects of PPC damage on tasks that involve forming associations between multiple sensory stimuli. In Experiment 1, sham or electrolytic lesions of the PPC were made before rats were tested on a three-phase sensory preconditioning task. During the first phase, half of the training trials consisted of pairings of an auditory stimulus followed by a light. During the other trials, a second auditory stimulus was presented alone. In the next phase of training, the same light was paired with food, but no auditory stimuli were presented. During the final phase of the procedure both auditory stimuli were presented in the absence of reinforcement during a single test session. As is typically observed during the test session, control rats exhibited greater conditioned responding to the auditory cue that was previously paired with light compared to the unpaired cue. In contrast, PPC-lesioned rats responded equally to both auditory cues. In Experiment 2, PPC-lesioned and control rats were trained in a compound feature negative discrimination task consisting of reinforced presentations of a tone-alone and non-reinforced simultaneous presentations of a light-tone compound stimulus. Control rats but not rats with damage to the PPC successfully learned the discrimination. Collectively, these results support the idea that the PPC contributes to relational learning involving multimodal sensory stimuli, perhaps by regulating the attentional processing of conditioned stimuli.

## Introduction

The posterior parietal cortex (PPC), along with the retrosplenial cortex (RSP), provides the primary source of polymodal visuo-spatial information to the postrhinal cortex (POR), which in turn has reciprocal connections with entorhinal cortex and discrete regions of the hippocampus (Burwell and Amaral, 1998a,b; Burwell, 2000; Furtak et al., 2007). Thus, PPC is ideally situated to contribute significantly to hippocampal-dependent functions, such as relational or configural learning, which involve processing information about multiple stimuli (Rudy and Sutherland, 1989, 1995; Eichenbaum and Cohen, 2001; Ryan et al., 2010). Indeed, disconnecting the PPC from hippocampus has been shown to impair performance during an object-place paired associates task (Rogers and Kesner, 2007). However, few studies have examined the contribution of PPC to other, non-spatial forms of relational learning.

The present study used a sensory preconditioning task and a compound feature negative discrimination task to examine the role of the PPC in non-spatial relational learning involving multimodal sensory stimuli. These tasks were chosen for several reasons. First, previous studies from our laboratory demonstrated that damage to RSP impairs performance on both tasks (Keene and Bucci, 2008b; Robinson et al., 2011), thus, given the similar anatomical connections of PPC and RSP, we were interested in comparing the effects of PPC and RSP lesions in these forms of learning. Moreover, the effects of hippocampal damage or lesions of areas of rhinal cortex have also been tested in these paradigms (Nicholson and Freeman, 2000; Ward-Robinson et al., 2001; Talk et al., 2002; Campolattaro and Freeman, 2006a,b). A second reason was that these paradigms involve learning about relationships between *phasic* stimuli, which is a particularly important feature because it has previously been shown that PPC damage does not impair contextual fear conditioning (Keene and Bucci, 2008a), which requires the formation of associations between multiple *static* environmental stimuli.

The sensory preconditioning task (Experiment 1), adapted from Brogden (1939) was conducted in three phases. During the “preconditioning” phase, an auditory stimulus (e.g., a tone) was presented and followed immediately by a light on half of the trials. During the other half of the trials another auditory stimulus (e.g., white noise) was presented alone. No reinforcement was delivered during this phase. During the subsequent “conditioning” phase, the same light was presented and followed by food reward. Finally, during the “post-conditioning” phase, a single test session assessed conditioned responding (food cup behavior) in response to each of the auditory stimuli by presenting them alone. If rats formed an association between the auditory stimulus that was paired with light during preconditioning, and if the significance of this relationship was updated after light → food conditioning, then food cup behavior was predicted to be particularly high in response to the paired stimulus, reflecting relational learning (Holland and Ross, 1983; Leising et al., 2007; Blaisdell et al., 2009; Robinson et al., 2011).

In Experiment 2, another set of PPC-lesioned rats was trained in a compound feature negative discrimination paradigm. Rats received two types of training trials: during reinforced trials, a tone was presented for 10 s and immediately followed by food reward; on non-reinforced trials, a light was presented concurrently with the tone and no food was delivered. Normal rats typically learn to approach the food cup in anticipation of receiving the food reward on tone-alone trials but withhold responding during light-tone simultaneous compound trials, indicating that rats form a relationship between the light and tone to inhibit responding (Chan et al., 2003).

## Materials and methods

### Subjects

Male Long Evans rats weighing ~225 g were obtained from Harlan Laboratories (Indianapolis, IN). Rats were housed individually and allowed seven days to acclimate to the vivarium with food available *ad libitum* (Purina standard rat chow; Nestle Purina, St. Louis, MO). Subsequently, rats were handled for 2 min per day for three days and weighed daily to establish baseline body weights, which were then gradually reduced to 85% of baseline over a seven-day period. Throughout the study, rats were maintained on a 14:10 light-dark cycle and monitored and cared for in compliance with the Association for Assessment and Accreditation of Laboratory Animal Care guidelines and the Dartmouth College Institutional Animal Care and Use Committee. All efforts were made to minimize discomfort for the animals.

### Surgery

Rats were anesthetized with isoflurane gas (1.5–3% in oxygen) and placed in a Kopf stereotaxic apparatus. To make bilateral electrolytic lesions of the PPC (Experiment 1, *n* = 11; Experiment 2, *n* = 8), the skin was retracted and small holes were drilled through the skull at the following eight locations (in mm): AP, −3.7, −4.7; ML, ±2.5, ±3.7; DV (from skull), −1.6, −1.8. These coordinates were based on previous reports that targeted the PPC (Bucci and Chess, 2005; Keene and Bucci, 2008a; Kesner, 2009) with boundaries based on thalamic and cortical connections (Chandler et al., 1992; Reep et al., 1994; Bucci et al., 1999; Paxinos and Watson, 2007). An electrode that was epoxy-coated except for the tip was lowered into each coordinate and a 2.5-mA current was passed through the tip for 15 s per lesion site. The needle was slowly retracted after the current was delivered and the skin was stapled together with wound clips. Electrolytic lesions were used to provide control over the extent of damage, which was an important factor in this study given the close proximity of RSP, which also provides visuo-spatial input to the medial temporal lobe (Burwell and Amaral, 1998a; van Groen and Wyss, 1990, 1992, 2003) and because we wanted to directly compare the effects of PPC-lesions to RSP lesions that were carried out using electrolytic methods in prior studies (Keene and Bucci, 2008a,b). Control rats (Experiment 1, *n* = 15; Experiment 2, *n* = 8) received sham lesions consisting of a craniotomy and shallow, non-puncturing burr holes to minimize damage to underlying cortex. Rats were allowed to recover for two weeks before behavioral training.

### Behavioral apparatus

#### Conditioning chambers

The behavioral apparatus was obtained from Med Associates Inc. (St. Albans, VT) and consisted of standard operant conditioning chambers (24 × 30.5 × 29 cm) connected to a computer and enclosed in sound-attenuating chambers (62 × 56 × 56 cm) outfitted with an exhaust fan to provide airflow and background noise (~68 dB). The operant chambers consisted of aluminum front and back walls, clear acrylic sides and top, and grid floors. A dimly illuminated food cup was recessed in the center of the front wall. A 2.8-W house light was mounted on the opposite wall and served as the visual stimulus. During stimulus presentation, the light flashed at a frequency of 2 Hz during preconditioning and conditioning. A speaker was located 15 cm above and to the right of the food cup and was used to present the tone (1500 Hz, 78 dB) and white noise (78 dB, Experiment 1 only) stimuli. A red, 2.8-W bulb was mounted on the ceiling of the sound-attenuating chamber to provide background illumination. A pair of infrared photocells was mounted just inside the food cup to detect head entries into the cup. Surveillance cameras located inside the sound attenuating chambers were used to monitor the rats' behavior.

#### Open field apparatus

Locomotor activity was assessed in an open field apparatus (43.2 × 43.2 × 30.5 cm) composed of Plexiglas walls and floor (Med Associates, Inc.). The chambers were equipped with 16 infrared photobeams that were arrayed 5.5 cm apart. Photobeam interruptions were recorded by a computer running custom Open Field Activity Monitoring software (Med Associates Inc.) that calculated the total distance traveled.

### Behavioral procedures

#### Experiment 1

**Figure 1:**
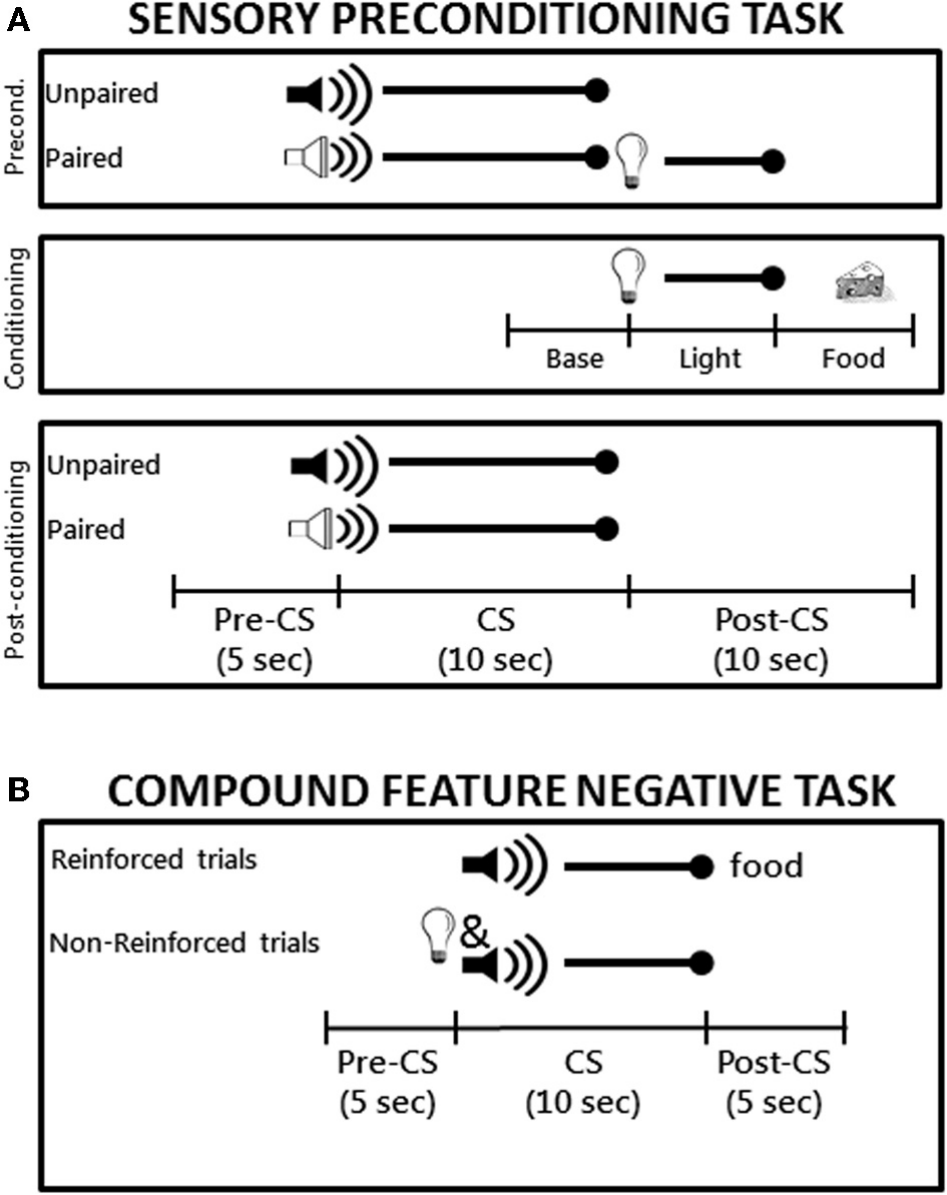
**(A)** Schematic diagram the sensory preconditioning task used in Experiment 1. The time line and epochs (Preconditioning: Baseline, Light, Food; Post-conditioning: Pre-CS, CS, Post-CS) noted on the bottom refer to the time periods used in the analyses described in the Materials and Methods. **(B)** Schematic diagram of the compound feature negative discrimination task used in Experiment 2. Rats were tested on a total of 8 daily conditioning sessions.

***Sensory preconditioning.*** A schematic diagram of the sensory preconditioning task is shown in Figure 1A. During the preconditioning phase, rats received four daily 64-min training sessions each consisting of 12 trials. On six of the trials, one of the auditory stimuli (the paired stimulus) was presented for 10 s and followed immediately by the 5-s flashing light stimulus. During the other six trials the other auditory stimulus (the unpaired stimulus) was presented alone for 10 s. The trials types were randomly intermixed with an average inter-trial interval (ITI) of 4.5 min and the use of the tone and white noise as the paired and unpaired auditory stimuli was counterbalanced across groups. During the conditioning phase, rat received seven daily 64-min conditioning sessions each of which consisted of eight presentations of the flashing light (5 s in duration, average ITI of 8 min) followed immediately by delivery of two 45-mg food pellets (Noyes, New Brunswick, NJ). Note that neither auditory stimulus was presented during the conditioning phase. Finally, the post-conditioning phase consisted of a single test session during which each of the two auditory stimuli were presented alone (six times each) in separate intermixed trials (78-min session).

***Locomotor activity.*** After the completion of the post-conditioning phase, rats were placed individually in a novel open field chamber for 10 min to test for potential activity changes induced by PPC-lesions.

#### Experiment 2

A schematic diagram of the compound feature negative discrimination task is shown in Figure 1B. Rats were magazine trained during a single 64 min session during which two 45-mg food pellets were randomly delivered 16 times. Training took place over eight daily sessions that lasted 64 min each and included 16 trials of two types. Rats received four trials per session consisting of a 10-s presentation of the tone followed immediately by delivery of two 45-g food pellets. For the other 12 trials, the panel light was presented simultaneously with the tone (10 s) and no food was delivered on these trials. The two trial types occurred randomly during the session and the order of trials differed on each day. The variable ITI averaged 4 min during magazine training and conditioning sessions.

### Behavioral observations and data analysis

#### Experiment 1

***Sensory preconditioning.*** Breaks in the photobeam located across the entry of the food cup were monitored by the computer. The amount of time the beam was broken served as the measure of conditioned food cup behavior. During conditioning, beam break data was collected during the 5-s period prior to onset of the visual stimulus (“Baseline” epoch), during the 5-s presentation of the visual stimulus (“Light” epoch) and during the 5 s period in which food was delivered (“Food” epoch) as shown in the schematic in Figure 1A. Data from the Light and Food epochs were subjected to repeated measures analysis of variance (rmANOVA) with Group (control, PPC-lesion) as the between-subjects variable and Session (1–7) as the within-subjects variable.

During the post-conditioning test session, beam break data was collected during three epochs: the 5-s period prior to onset of the auditory conditioned stimuli (“Pre-CS” epoch), the 10-s period during presentation of the auditory stimuli (“CS” epoch), and during the 10-s period following presentation of the auditory stimuli (“Post-CS” epoch). Data from the CS and Post-CS epochs were subjected to rmANOVA with Group (control, PPC-lesion) as the between-subjects variable and Trial Type (Paired stimulus, Unpaired stimulus) as the within-subjects variable. Significant main effects were followed up with appropriate pair-wise comparisons (two-tailed *t*-tests). Data from the Post-CS epoch were particularly important to analyze because this period corresponded to the time that the light was presented after the auditory stimulus in the preconditioning phase and also to the time that food would have been presented during light → food conditioning in the conditioning phase. An additional comparison of the strength of sensory preconditioning between the control and lesioned groups was carried out on the Post-conditioning session data by calculating a difference score, defined as the amount of responding observed during the Post-CS period following presentation of the paired auditory stimulus divided by the sum of the Post-CS responding observed following each of the auditory stimuli. Using one-sample *t*-tests, the resulting values for each group were compared to an expected value of 50% (i.e., chance), which would indicate no sensory preconditioning.

***Locomotor activity.*** Open field activity data was analyzed with rmANOVA with Group (control, PPC-lesion) as the between-subjects variable and Epoch (1-min periods) as the within-subjects variable. An alpha level of 0.05 was used in all analyses.

#### Experiment 2

***Compound feature negative discrimination task.*** As in Experiment 1, breaks in the photobeam located across the entry of the food cup were monitored by the computer and the amount of time the beam was broken served as the measure of conditioned food cup behavior. As demonstrated in previous studies (Holland et al., 1999; Keene and Bucci, 2008b), rats typically exhibit increasing levels of responding on both trial types for the first few sessions and do not discriminate between them. Indeed, the main data of interest are the levels of conditioned responding that are achieved when rats have reached stable performance levels on both types of trials. Thus, the data from the last two sessions were averaged and subjected to rmANOVA with Group (control, PPC-lesion) as the between-subjects variable and Trial Type (reinforced, non-reinforced) as the within-subjects variable. Analyses were conducted during the 5-s period prior to CS onset (Pre-CS responding), during presentation of the CS, and during the 5 s after the CS was turned off (Post-CS responding). Significant main effects were followed up with appropriate pair-wise comparisons (two-tailed *t*-tests). In addition, a difference score was calculated by subtracting responding during non-reinforced trials from responding during reinforced trials during the last two sessions. This was used to assess the magnitude of the discrimination in each group. An alpha level of 0.05 was used in all analyses.

### Lesion verification and analysis

**Figure 2:**
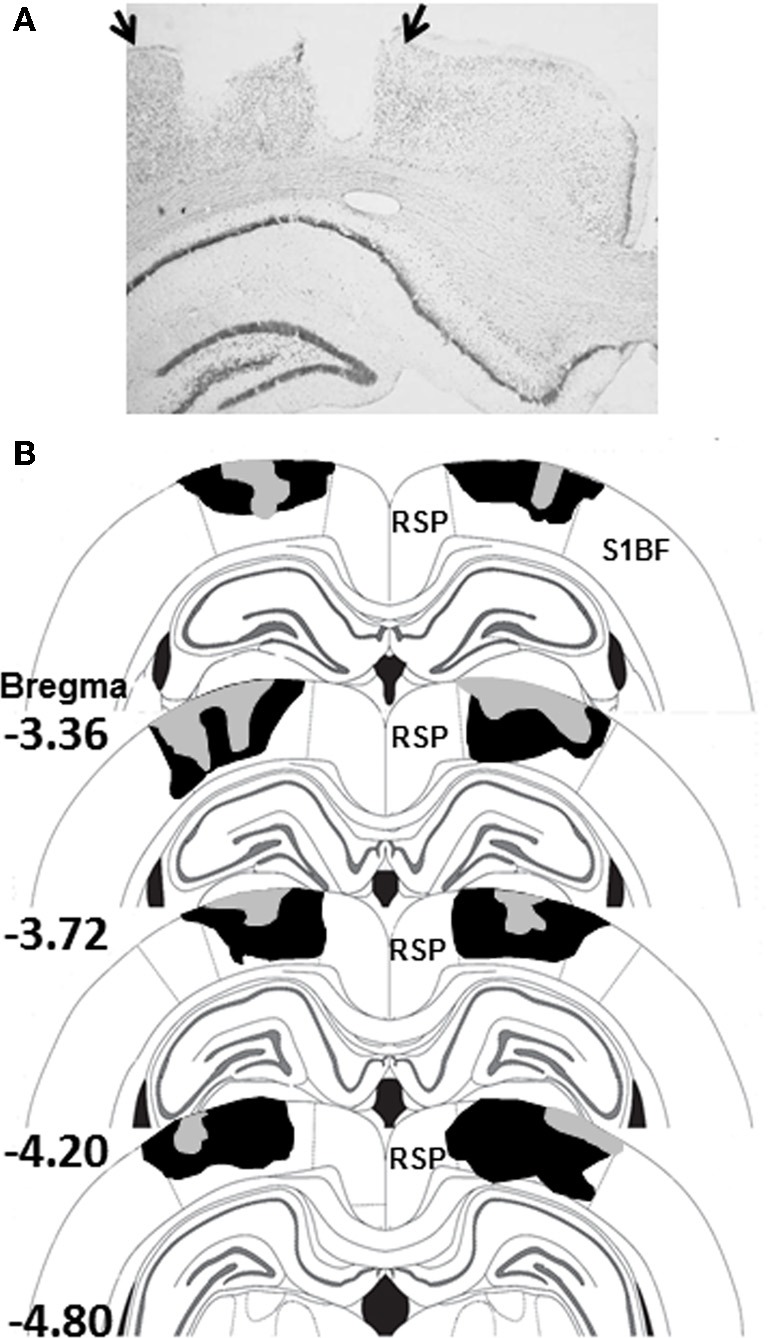
**(A)** Photomicrograph of a PPC-lesion illustrating typical damage to PPC on one side of the brain. The arrows indicate the boundaries of PPC. **(B)** Schematic diagram indicating the largest (black) and smallest (gray) lesions of PPC in Experiment 1 (adapted from Paxinos and Watson, 2007). Abbreviations: RSP, restrosplenial cortex; S1BF, somatosensory cortex barrel fields.

After the behavioral procedures were completed, rats were deeply anesthetized with an overdose of pentobarbital sodium and phenytoin sodium (Euthasol, Virbac Animal Health, Fort Worth, TX) and transcardially perfused with 0.9% saline for 5 min, followed by 10% buffered formalin. Brains were sectioned on a freezing microtome (60 μm) and Nissl-stained using thionin. For each animal, coronal sections at four AP locations (from Bregma: −3.36, −3.72, −4.20, −4.80; see Figure 2B) along the rostrocaudal extent of the PPC were used to assess the amount of tissue damage. Using StereoInvestigator software (Version 9; Microbrightfield, Inc., Williston, VT) and a compound microscope (Axioskop I, Zeiss, Inc.), gross tissue damage as necrosis, missing tissue, or marked thinning of the cortex was identified. For each coronal section, areal measurements were obtained using the StereoInvestigator Cavalieri estimator probe with 50 μm grid spacing. Lesion size is expressed as the percentage of damage to the target region divided by the total area of the target region.

## Results

### Histology

Electrolytic damage to the PPC is displayed in the photomicrograph in Figure 2A and the largest and smallest of the 11 PPC-lesions from Experiment 1 are shown in Figure 2B. Bilateral PPC damage was observed in all rats and the average area of PPC damaged on each section analyzed in Experiment 1 was 49 ± 3% (range 36–69%). Minor unilateral damage to the RSP was observed in one animal and minor unilateral damage to somatosensory cortex was observed in two animals. Minor unilateral damage to the corpus callosum was observed in 4 animals. In Experiment 2, damage to the PPC in lesioned rats was similar to that observed in Experiment 1 and to previous studies from our lab (Keene and Bucci, 2008a). Bilateral PPC damage was observed in all rats and the average area of PPC damaged on each section analyzed was 48 ± 3% (range 33–60%).

### Behavior

#### Experiment 1

**Figure 3:**
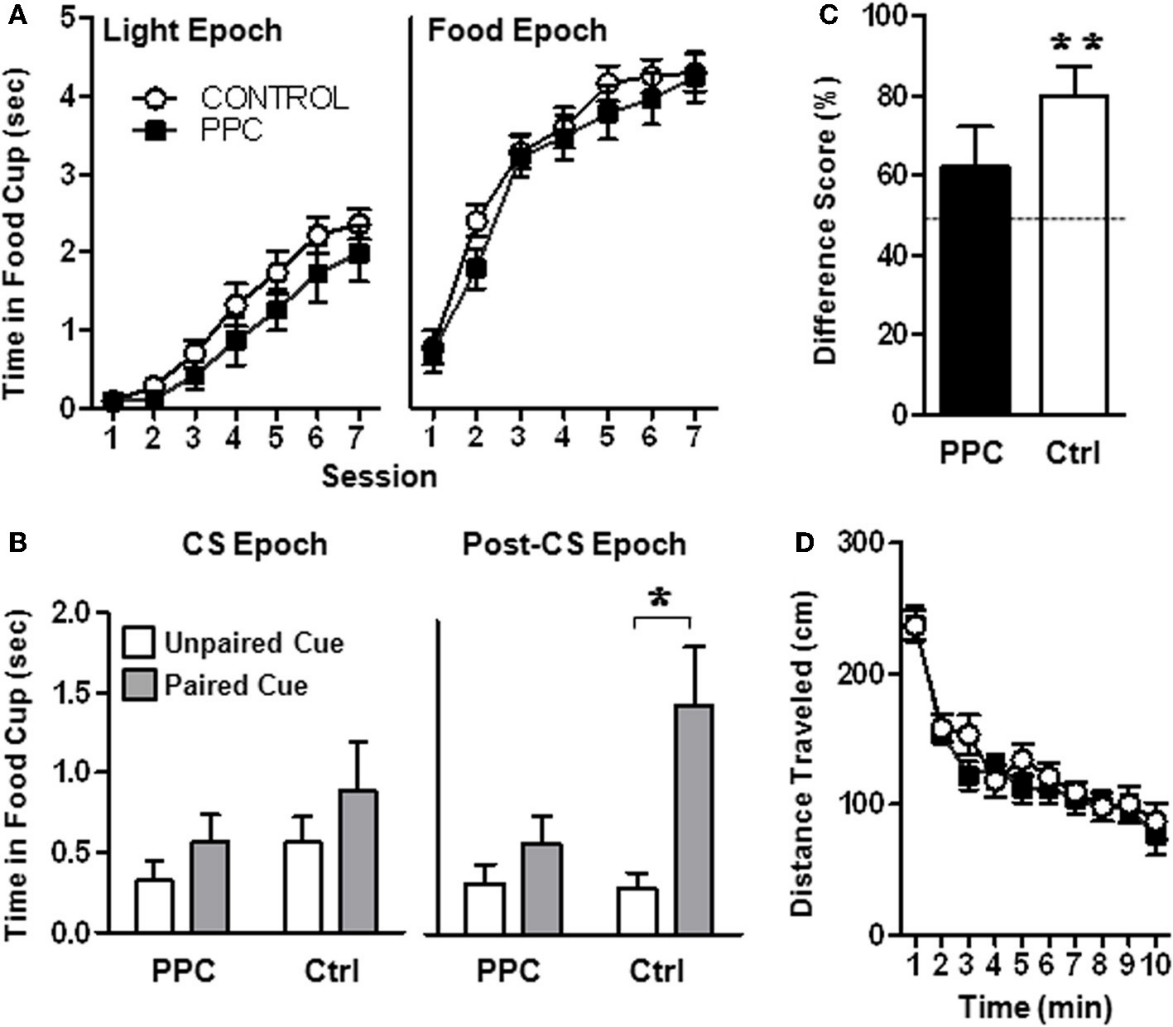
**Experiment 1.** PPC-lesioned rats exhibit impaired sensory preconditioning. **(A)** Food cup responding during the Light epoch (left panel) and during the Food epoch (right panel) during Phase 2 of the sensory preconditioning task. No group differences were observed, indicating that PPC damage did not affect light-food conditioning or food retrieval. **(B)** Food cup responding during the CS epoch (left panel) and the Post-CS epoch (right panel) following presentation of the two auditory stimuli during the post-conditioning phase. Control but not PPC-lesioned rats exhibited sensory preconditioning, evidenced by greater food cup responding during presentation of the auditory stimulus that was previously paired with the light compared to the unpaired auditory stimulus during the Post-CS epoch. **(C)** Food cup behavior difference scores calculated from the Post-CS epoch during the test session. Control, but not PPC-lesioned rats exhibited difference scores significantly different from 50%, indicating that during the Post-CS epoch, more time was spent with the snout in the food cup on paired stimulus trials compared to unpaired stimulus trials. **(D)** Open field activity demonstrating that the distance traveled by PPC-lesioned rats (*n* = 11) did not differ from that of control rats (*n* = 15) and that both groups similarly habituated to the open-field over time. Data are mean ± standard error. ^*^Indicates a significant (*p* < 0.05) difference in food cup behavior by control rats on unpaired vs paired (sensory preconditioned) trials. ^**^Indicates a significant (*p* < 0.05) difference from chance (50%).

***Sensory preconditioning.*** As shown in Figure 3A (left panel), as training progressed during the conditioning phase, rats in both groups exhibited increased food cup behavior during presentation of the light (Light epoch). This was confirmed by a rmANOVA that revealed a significant main effect of Session [*F*_(6, 144)_ = 55.0, *p* < 0.001]. The main effect of Group and the Group X Session interaction were not statistically significant (*p*s > 0.2), indicating that control and PPC-lesioned rats comparably learned the association between the light and food. Similarly, analysis of data from the Food epoch (Figure 3A, right panel) revealed that both groups increased food cup responding across training sessions [*F*_(6, 144)_ = 90.5, *p* < 0.001]. The main effect of Group and the Group X Session interaction were not statistically significant (*p*s > 0.2), suggesting that control and PPC-lesioned rats were comparably motivated to retrieve food.

The critical test session data collected during post-conditioning phase is illustrated in Figure 3B. A rmANOVA that compared the food cup behavior of control and PPC-lesioned rats during the Post-CS epoch revealed a significant main effect of Trial Type [*F*_(1, 24)_ = 11.25, *p* < 0.01] and a significant Trial Type X Group interaction [*F*_(1, 24)_ = 4.68, *p* < 0.05]. Importantly, there was no main effect of Group (*p* > 0.1) indicating that control and PPC-lesioned rats exhibited similar overall levels of food cup responding. Subsequent paired *t*-tests on test session data from the Post-CS epoch (Figure 3B, right panel) revealed that control rats spent more time in the food cup on trials in which the paired auditory stimulus was presented compared to trials in which the unpaired auditory stimulus was presented [*t*_(14)_ = −3.4, *p* < 0.01], indicating that control rats formed a stimulus–stimulus association during the preconditioning phase that was updated following the light-food conditioning phase. Unlike control rats, animals with PPC damage exhibited similar food cup responding during the Post-CS epoch regardless of whether the paired or unpaired auditory stimulus was presented. The rmANOVA conducted on the CS-epoch data did not reach statistical significance (Figure 3B, left panel; *p*s > 0.1).

A complementary analysis was conducted using difference scores (calculated by dividing the time spent in the food cup during the Post-CS epoch following presentation of the paired stimulus by the sum of the Post-CS responding observed following presentation of each auditory stimulus during the critical post-conditioning-test session), as presented in Figure 3C. Control rats had a mean difference score that was significantly higher than 50% [*t*_(14)_ = 4.1, *p* < 0.001; mean = 80.1 ± 7.2%] but PPC-lesioned rats did not (*p* > 0.2; mean = 61.9 ± 10.3%). These data are consistent with the results of the primary rmANOVA above in suggesting that PPC damage impaired sensory preconditioning.

***Locomotor activity.*** Assessment of open field activity (Figure 3D) revealed that there were no differences in total activity or habituation to the open field (*p*s > 0.6) between control and PPC-lesioned rats.

#### Experiment 2

**Figure 4:**
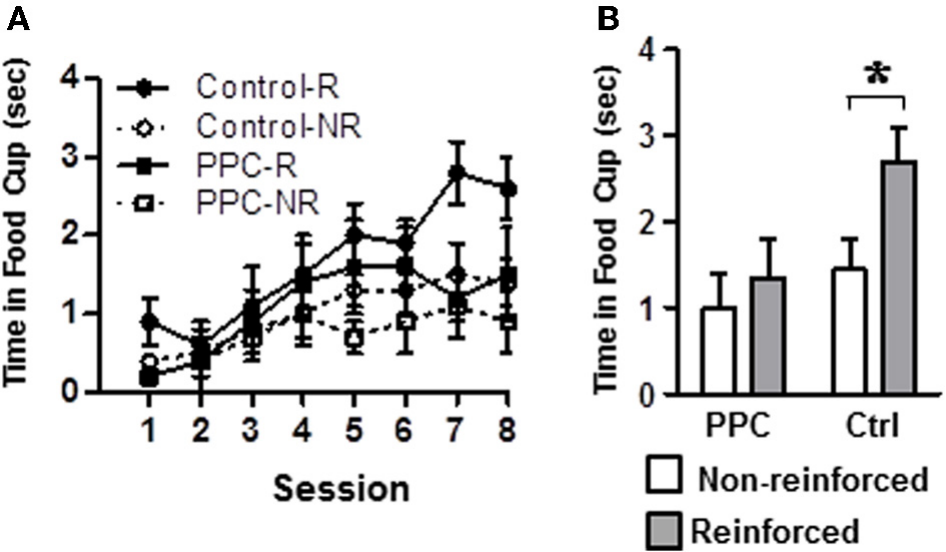
**Experiment 2.** Damage to PPC impaired learning on the compound feature-negative discrimination task. **(A)** Conditioned food cup behavior following presentations of the tone-alone (reinforced) and following presentations of the compound stimulus (light-tone, non-reinforced) across all eight conditioning sessions. **(B)** Combined data from conditioning sessions 7 and 8 during which stable performance is typically observed. Data are mean ± standard error. Abbreviations: R, reinforced stimulus; NR, non-reinforced stimuli. ^*^Indicates a significant (*p* < 0.05) difference in food cup behavior by control rats on R verses NR trials.

Figure 4A illustrates conditioned responding during presentations of the tone and light-tone compound stimuli across all eight sessions of the compound feature negative discrimination task. Figure 4B displays average conditioned responding during the last two sessions, when stable performance is typically observed. A rmANOVA on the data from the last two sessions revealed a significant main effect of Trial Type [*F*_(1, 14)_ = 24.1, *p* < 0.001] and a significant Trial Type X Group interaction [*F*_(1, 14)_ = 7.7, *p* < 0.02], but no significant effect of Group, indicating that the groups differed in their ability to discriminate between the two trials, but not in their overall responding. Subsequent analysis revealed that control rats exhibited significantly more food cup behavior during reinforced trials compared to non-reinforced trials [*t*_(7)_ = 5.4, *p* < 0.001]. In contrast, PPC-lesioned rats exhibited comparable levels of responding on both trial types [*t*_(7)_ = 1.5, *p* > 0.2]. Additional comparisons indicated that on reinforced trials control rats spent more time in the food cup than did PPC-lesioned rats [*t*_(14)_ = 2.4, *p* < 0.03]. There was no significant group difference in responding during the non-reinforced trials [*t*_(14)_ = 0.9, *p* > 0.4]. The magnitude of the discrimination, as assessed by the difference in responding on reinforced and non-reinforced trials, also differed significantly between control and PPC-lesioned rats [*t*_(14)_ = 2.8, *p* < 0.02]. The difference scores for control and PPC-lesioned rats were 1.3 ± 0.2 s and 0.3 ± 0.2 s, respectively, indicating that PPC-lesions impaired the ability to discriminate between the two trials types, consistent with the findings of the primary ANOVA above.

Group differences in food cup behavior exhibited during the 5-s period prior to CS onset (i.e., pre-CS responding) were analyzed to test for differences in baseline responding. The amount of food cup behavior exhibited prior to the start of a trial was very low and did not differ between control and PPC-lesioned rats [*F*_(1, 14)_ = 0.02, *p* > 0.9]. The mean amount of time spent with the snout in the food cup during the pre-CS period was 0.4 ± 0.1 s for both groups. Responding during the 5-s period immediately after the tone was turned off and food was delivered (i.e., Post-CS responding) was also examined to assay for potential group differences in retrieving food. A rmANOVA indicated that Post-CS responding was comparable between control and PPC-lesioned rats [*F*_(1, 14)_ = 2.7, *p* > 0.1]. The mean time spent in the food cup during the Post-CS epoch was 4.3 ± 0.2 s and 3.9 ± 0.2 s for control and PPC-lesioned rats, respectively.

## Discussion

The present study tested the effects of PPC damage on two non-spatial tasks that involve encoding information about multiple phasic sensory stimuli. In Experiment 1, sensory preconditioning occurred in control but not PPC-lesioned rats. In Experiment 2, PPC damage impaired the ability of rats to learn a conditional discrimination between a reinforced single stimulus (e.g., a tone) and a non-reinforced compound stimulus (e.g., tone and light).

One objective of the present study was to compare the effects of damage to the PPC with previous observations following damage to the RSP. Of particular relevance, a recent series of studies demonstrated that RSP-lesioned rats were impaired in their ability to solve a variety of tasks that involved the formation of stimulus–stimulus associations regardless of whether stimuli are presented simultaneously (Keene and Bucci, 2008b), serially, or in the absence of reinforcement (Robinson et al., 2011). In addition, RSP damage also impairs contextual fear conditioning, which requires the formation of associations between multiple *static* environmental cues (Keene and Bucci, 2008a). These findings are consistent with those of Gabriel and colleagues who demonstrated that neurons in the posterior cingulate cortex of rabbits (thought to be comparable to RSP in rats) are sensitive to the formation of associations between a tone and different contexts in an approach/avoidance discrimination task (Freeman et al., 1996; Smith et al., 2004). Collectively, these data support the notion that RSP has a general role in forming stimulus–stimulus associations, regardless of whether the cues are static or phasic. Therefore, RSP may be essential for binding cues together to facilitate learning about behaviorally relevant stimuli.

Thus, one interpretation of the present results is that PPC damage also produces a general impairment in the ability to form stimulus–stimulus associations. Importantly, however, a shortcoming of this interpretation is that unlike RSP damage, PPC damage does not impair contextual fear conditioning (Keene and Bucci, 2008a). Perhaps rather than having a general role in the formation of stimulus–stimulus associations, the PPC contributes to relational learning situations in which stimuli are phasic and therefore more likely to garner attention compared to static cues. This possibility is consistent with a substantial literature indicating that PPC neurons fire transiently during the onset of a stimulus, or in response to a change in a stimulus, but stop firing during sustained presentation of a stimulus (Mountcastle et al., 1975; Robinson and Goldberg, 1978; Bushnell et al., 1981). Similarly, the PPC has repeatedly been shown to mediate increases in attention that are necessary for processing changes in the meaning of individual stimuli or changes in the relationships between stimuli (Bucci et al., 1998; Fox et al., 2003; Bucci and Chess, 2005; Bucci and Macleod, 2007; Maddux et al., 2007; Bucci, 2009). With respect to the sensory preconditioning task used in the present study, contemporary learning theories (Pearce and Hall, 1980; Wilson et al., 1992) maintain that attentional processing would be high because the light is first non-reinforced during the preconditioning phase, but then followed by food reward during the conditioning phase. Thus, if PPC mediates relational learning about phasic stimuli with ambiguous or changed meanings, then PPC-lesioned rats would be impaired in their ability to update the significance of the tone-light relationship and thus subsequently fail to discriminate between the two auditory stimuli during the post-conditioning test session.

A potential flaw in this explanation lies in the fact that the meaning of the light also changed (i.e., first non-reinforced, then later paired with food), which would lead to the prediction that PPC-lesioned rats would also be impaired in learning the light-food relationship. Indeed, this was true in similar study (Bucci and Chess, 2005), but not in the present study. However, a key difference may be that in the study by Bucci and Chess (2005), the light was always presented alone in the non-reinforced phase, rather than being preceded by a tone (present study). Indeed, it has been suggested that pairing the tone and light in the sensory preconditioning paradigm may “protect” the light from latent inhibition, leading to intact learning in the conditioning phase of the sensory preconditioning task (Pfautz et al., 1978).

A similar attentional account may also explain the deficits observed in the compound feature negative discrimination task in Experiment 2. In that paradigm, tone-alone trials were always reinforced, while light-tone trials were always non-reinforced. Although there was no change in the meaning of the stimuli as there was in Experiment 1, it is important to note that the procedure used in the compound feature negative discrimination task amounts to a partial reinforcement paradigm, in that the tone is only reinforced on a subset of trials. As described previously, partial reinforcement contingencies typically enhance attentional processing of conditioned stimuli (Pearce and Hall, 1980). The conceptualization that PPC is particularly involved in processing changes in stimuli as described above also may explain the absence of impairment in contextual fear conditioning (Keene and Bucci, 2008a). Indeed, one difference between the conditioning tasks used here and contextual fear conditioning is that the conditioned stimuli in the latter paradigm are static cues. In other words, the contextual stimuli in the fear conditioning task are always present, regardless of whether footshock is delivered. In contrast, the tasks used in the present study involved phasic cues, which are only presented for short periods of time. Future studies could investigate the contribution of PPC to attentional processing during relational learning by systematically manipulating attentional load in permutations of the tasks used here.

Evidence that PPC contributes to relational learning informs the question of how different cortico-hippocampal circuits contribute to medial temporal lobe dependent learning and memory. Hippocampal damage has been shown to impair performance in a serial feature negative discrimination task (Holland et al., 1999) but spares learning a compound feature negative discrimination (Solomon, 1977; Chan et al., 2003). In contrast, PPC-lesions impair compound feature negative discrimination (Experiment 2). In addition, hippocampal damage has been shown to have an equivocal effect on sensory preconditioning, with some studies reporting deficits (Talk et al., 2002) and others observing no effects (Ward-Robinson et al., 2001). These findings support the notion that PPC may have a distinct role from the hippocampus during relational learning. This is consistent with recent theories delineating functional distinctions of a medial temporal lobe system believed to support episodic memory (Davachi, 2006; Diana et al., 2007; Eichenbaum et al., 2007). It is also noteworthy that damage to perirhinal cortex (PER) impairs sensory preconditioning (Nicholson and Freeman, 2000), compound feature negative discrimination (similar to Keene and Bucci, 2008b) and compound feature positive discrimination while having no effect on learning a serial feature positive discrimination (Campolattaro and Freeman, 2006a,b). Based on these findings, it was suggested that PER may play a role in resolving ambiguity in discriminations with overlapping stimulus elements (Campolattaro and Freeman, 2006a,b). Thus, it is possible that PER, PPC, and RSP contribute to complex learning paradigms by resolving stimulus ambiguity for overlapping stimulus elements, by allocating attention to changes in meaningful cues and by forming or mediating associations between multiple stimuli, respectively. These proposed functions of PER and RSP are consistent with another recent study that found unique contributions of CA1 and dorsocaudal medial entorhinal (dcMEC) cortex to the disambiguation of overlapping experiences (Lipton et al., 2007). Critical to the present discussion, this study establishes that nearby cortical structures (i.e., dcMEC) make important and distinct contributions to hippocampal function in resolving ambiguity for closely related or overlapping experiences. This idea, along with the present findings, provides an intriguing avenue for future research regarding the unique contributions of closely related brain areas such as PPC, PER, RSP, and the hippocampus.

The PPC is strongly connected with visuo-spatial areas (Miller and Vogt, 1984; Kolb and Walkey, 1987; Reep et al., 1994) and therefore, it is possible that the observed deficits in the present study could merely be due to an inability to process visual stimuli. Similarly, the use of electrolytic techniques may have damaged fibers of passage from these areas. This does not seem likely, however, since conditioning to the light was comparable in the control and PPC-lesioned groups during the sensory preconditioning task. It is also unlikely that alterations in motivation levels can explain the deficits in either task, since PPC-lesioned rats were no different from controls in approaching the food cup and consuming food when was delivered. Likewise, the impairments in conditioned responding during the test phase of the sensory preconditioning task or during the compound feature negative discrimination task were not due to lesion-induced changes in locomotor activity. Instead, the present findings support the notion that PPC contributes to hippocampal-dependent forms of relational learning, perhaps by regulating attentional processing of specific cues. In addition, these data are consistent with the notion that separate components of cortico-hippocampal circuits may have discernible roles in medial temporal lobe related behavior.

### Conflict of interest statement

The authors declare that the research was conducted in the absence of any commercial or financial relationships that could be construed as a potential conflict of interest.
